# Enhanced Persistent Luminescence from Cr^3+^-Doped ZnGa_2_O_4_ Nanoparticles upon Immersion in Simulated Physiological Media

**DOI:** 10.3390/nano15030247

**Published:** 2025-02-06

**Authors:** Clement Lee, David Park, Wai-Tung Shiu, Yihong Liu, Lijia Liu

**Affiliations:** Department of Chemistry, Western University, 1151 Richmond Street, London, ON N6A 5B7, Canada; hlee752@uwo.ca (C.L.); dpark68@uwo.ca (D.P.); wshiu@uwo.ca (W.-T.S.); yliu3465@uwo.ca (Y.L.)

**Keywords:** Cr^3+^-doped zinc gallate, persistent luminescence nanoparticles, defect passivation, physiological media

## Abstract

Near-infrared persistent luminescence (PersL) nanoparticles (NPs) have great potential in biomedical applications due to their ability to continuously emit tissue-penetrating light. Despite numerous reports on the distribution, biological safety and other consequences of PersL NPs in vitro and in vivo, there has been a lack of studies on the optical properties of these NPs in the physiological environment. In light of this, we investigated the effects of short-term immersion of the prominent Cr^3+^-doped ZnGa_2_O_4_ (CZGO) NPs in a simulated physiological environment for up to 48 h. This paper reports the changes in the structural and optical properties of CZGO NPs after their immersion in a phosphate-buffered saline (PBS) solution for pre-determined time intervals. Interestingly, the luminescence intensity and lifetime noticeably improved upon exposure to the PBS media, which is unusual among existing nanomaterials explored as bioimaging probes. After 48 h of immersion in the PBS solution, the CZGO NPs were approximately twice as bright as the non-immersed sample. X-ray spectroscopic techniques revealed the formation of ZnO, which results in an improvement in observed luminescence.

## 1. Introduction

Near-infrared (NIR) emitting nanoparticles (NPs) are aspiring materials for biomedical imaging due to their emittance in the tissue transparency window (650–1800 nm) [1]. Optical imaging performed using NPs enables a non-invasive approach of dynamically monitoring disease progression, identifying pathological markers or studying the pharmacokinetics after drug administration [2,3,4]. However, bioimaging using conventional probes (e.g., organic dyes, inorganic quantum dots, etc.) requires constant excitation and cannot overcome the fundamental barrier of tissue autofluorescence [5]. This unwanted interference lowers the signal-to-noise ratio and can compromise diagnosis resolution, limiting existing applications which demand a higher degree of precision. Consequentially, several autofluorescence-free modalities have been developed, ranging from the use of genetically engineered bioluminescent agents to organic afterglow imaging [6,7]. One alternative method to avoid autofluorescence is to use NIR-emitting persistent luminescence (PersL) NPs to perform bioimaging [8,9,10]. Unlike typical optical probes, PersL materials can emit light for a prolonged duration, and their mechanism of light emittance is self-sustained, meaning continuous excitation from an external source is not necessary [11].

ZnGa_2_O_4_ is a wide band gap (~5 eV) semiconductor, and it has been investigated for various applications such as catalysis and photonics [12,13]. It has an AB_2_O_4_ spinel structure, where Zn^2+^ is located at the A site of a tetrahedral symmetry, and Ga^3+^ occupies the octahedral B site in the lattice. Since the ionic radii of Zn^2+^ and Ga^3+^ are similar, a small portion (~3%) of Zn^2+^ and Ga^3+^ exchange their site of occupancy, resulting in an unusual antisite defect [14]. Upon doping with various transition metals or lanthanides, ZnGa_2_O_4_ becomes luminescent; the electrons excited from the dopant or the valence band of ZnGa_2_O_4_ can undergo radiative decay through the dopant site. The antisite defects act as electron traps, extending the luminescence decay lifetime and producing PersL. For example, using Mn^2+^ as a dopant results in a green afterglow, which can be used for “glow-in-the-dark” objects, while the use of Cr^3+^ as a dopant leads to NIR emission, which can penetrate tissue and can be used for bioimaging [15,16]. To date, Cr^3+^-doped ZnGa_2_O_4_ (CZGO) has been one of the most promising PersL materials employed for biological applications [17]. In contrast to other NIR-emitting PersL materials, CZGO-based phosphors can be relatively easy to synthesize as nanosized particles under mild conditions, and their PersL can be produced by various excitation sources (e.g., UV light, sunlight, NIR laser and X-ray) [14,17] and their emission properties can be readily tuned by adjusting certain synthesis parameters [18,19].

In the past, there have been several in-depth investigations on the applicability of CZGO NPs after successful integration into mice [20,21,22]. In 2018, Sun et al. examined the in vivo toxicological effects of intravenously injecting CZGO NPs into mice. They reported no significant toxicities in the mice for 60 days, as indicated by their hematological and histological analyses. Throughout this duration, the NPs had accumulated in the liver of the mice, where their persistent emission remained detectable [20]. In the same year, Liu et al. studied the in vivo biodistribution of functionalized CZGO NPs within tumour-bearing mice after oral and subcutaneous administration [21]. They discovered the accumulation of CZGO NPs at the target tumour sites, with maximum luminescence at the tumour sites at 8 h. From tracking their PersL, the authors discovered that most of the CZGO NPs were either excreted through the renal system or metabolized in the intestines. A more recent study published in 2024 by Cai et al. studies the effects of in vivo imaging by introducing Sn^4+^ as a co-dopant to CZGO NPs [22]. Their results at 4 h revealed a 3-fold signal enhancement. As represented in a multitude of studies, most of the literature discussing the use of CZGO-based NPs has a strong focus on discovering methods for improving material design [18,22,23] or the efficacy of the intended application (e.g., tumour targeting [24,25,26], biosensing [27,28], bioimaging [29,30]). Conversely, there is a lack of reports focusing on the optical/structural changes to CZGO NPs after exposure to physiological environments.

It is also crucial to investigate the changes to the NPs upon short- and long-term immersion in physiological media. Any transformations to the NPs during exposure to biological conditions may result in unintended consequences upon in vivo application. For example, gold NPs (AuNPs), as one of the most investigated nanomaterials for biological use, were found to exhibit a loss in targeting ability when they were exposed to a physiological environment due to the accumulation of immune-related proteins on the surface [31]. In a separate study, González et al. discovered changes to photothermal capabilities depending on the morphology of AuNPs upon immersion in physiological fluids [32]. The study of NP immersion includes topics such as particle stability, changes to the particle surface, or efficiency changes as a result of structural and chemical changes, which may not always be identifiable when conducting application-focused research.

Notably, a study carried out by Lécuyer et al. reported the degradation of CZGO NPs in an artificial lysosomal fluid, which mimics the intracellular environment. Through their analysis, hydrothermally synthesized CZGO NPs first underwent surface hydroxylation to ensure they were dispersible in an aqueous solution. Their hydroxylated CZGO NPs were found to degrade to 50% after a one-month exposure to the lysosomal fluid, and 100% degradation was reported after three months [33]. Following this work, a one-year investigation was conducted in vivo using mice to study the long-term toxicity, retention and change to optical properties of CZGO [34]. They revealed no notable harm to the mice through histological and hematological evaluations after one year, suggesting its biological safety as a potential candidate for preclinical studies. Interestingly, in their lysosomal solution exposure experiment, although the luminescence from CZGO gradually decreased over 90 days, within the first 8 days, an enhanced luminescence intensity was observed. The authors attributed the luminescence enhancement to the suppression of surface defects, as there was no in-depth discussion on the change to the surface structure of the CZGO. Typically, the surface modification of nanoparticles is performed to improve their compatibility with a solvent or to add functionality to address targeted applications [35]. The initial luminescence enhancement upon exposure to the physiological media further benefits CZGO’s application in optical imaging. Therefore, a thorough understanding of how the solution interacts with the CZGO surface is desirable.

In Lécuyer’s work, which spanned from 3 months to 1 year, the use of artificial lysosomal fluid is crucial, as cellular uptake is a dominant contributor to NP degradation [36]. However, opsonization is often not immediate and many applications involving CZGO NPs take place within several hours to a few days, as demonstrated in several in vivo studies [20,21,37]. In light of this, herein, we immersed hydroxylated CZGO NPs in a phosphate-buffered saline (PBS) solution, the most conventional physiological medium used to simulate the bodily environment. We observed noticeable improvements to the optical properties, and we used several structure elucidation techniques to analyze and correlate the structural/optical changes to the CZGO NPs after their immersion in PBS.

## 2. Materials and Methods

### 2.1. Chemicals

The following reagents were of analytical grade and used as received. Zinc nitrate hexahydrate [Zn(NO_3_)_2_·6H_2_O, 98%], gallium nitrate hydrate [Ga(NO_3_)_3_·xH_2_O, 99.9%], sodium phosphate dibasic [Na_2_HPO_4_, ACS reagent grade] and hydrochloric acid [HCl, 37.0 wt%] were purchased from Sigma Aldrich (Oakville, ON, Canada). The sodium chloride [NaCl, ≥99.0%] and isopropanol were acquired from Fisher Chemical (Ottawa, ON, Canada). The chromium nitrate nonahydrate [Cr(NO)_3_·9H_2_O] was obtained from Alfa Aesar (London, ON, Canada). The ammonium hydroxide [NH_4_OH, 28.0 wt%] and potassium chloride [KCl, ≥99.0%] were purchased from Caledon Laboratories Ltd (Georgetown, ON, Canada). Lastly, the potassium phosphate monobasic [KH_2_PO_4_, ≥99.0%] was bought from BioShop Canada Inc. (Burlington, ON, Canada).

### 2.2. Preparation of PBS Solution

The PBS solution was prepared following an established protocol, which involves dissolving several inorganic salts in distilled water [38]. In short, 8 g of NaCl, 0.2 g of KCl, 1.44 g Na_2_HPO_4_ and 0.245 g of KH_2_PO_4_ were sequentially dissolved in 0.8 L of distilled water. The pH of the solution was measured to be 7.4 using the Fisherbrand pH Pen (London, ON, Canada). Lastly, an additional 0.2 L of distilled water was added to the above solution.

### 2.3. Synthesis of CZGO NPs

A hydrothermal synthesis procedure was used to synthesize the CZGO NPs [15]. In brief, 3.6 mmol of Zn(NO_3_)_2_·6H_2_O and 6 mmol Ga(NO_3_)_3_·xH_2_O were dissolved in 15 mL of deionized water. The solution underwent stirring for 10 min, and then 0.012 mmol of Cr(NO)_3_·9H_2_O was added. The pH of the above solution was adjusted to 9 using NH_4_OH, and the mixture was stirred for an additional 30 min [21,39]. The solution was transferred to a 25 mL Teflon-lined autoclave and placed in an oven for 10 h at a temperature of 220 °C. The crude product was obtained by centrifugation at 2000 rpm for 5 min and purified through several washes using 0.01 M HCl and isopropanol. The powder was dried overnight, then annealed in a muffle furnace at 900 °C for 1 h, with a ramp rate of 200 °C/h [18].

### 2.4. Surface Hydroxylation of CZGO NPs

**Figure 1:**
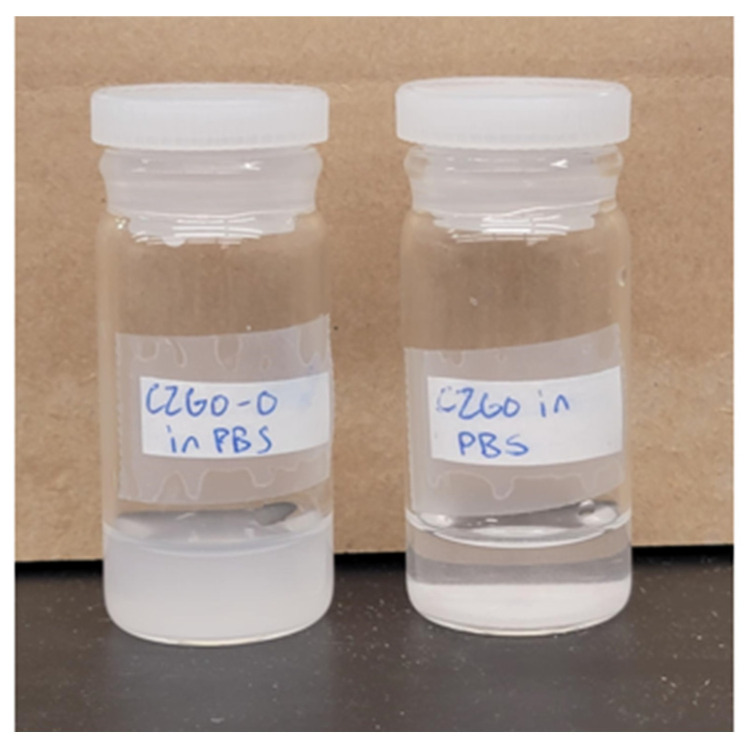
A photograph displaying the dispersion of CZGO-0 (**left**) and CZGO (**right**) in the phosphate-buffered saline (PBS) solution.

To improve the solubility of the CZGO NPs, surface hydroxylation was performed based on previously published protocols, with modifications [33]. First, 0.005 g of CZGO NPs was placed in a mortar, followed by 0.5 mL of 5 mM HCl. The powder was wet-ground for 15 min and placed in a beaker containing an additional 4.5 mL of 5 mM HCl. The solution was placed in the beaker and stirred overnight. The sample was collected by centrifugation at 8500 rpm for 10 min. The purification of the product was performed by washing the powder in isopropanol and water at 8500 rpm for 10 min twice. The hydroxylated sample is denoted as CZGO-0 (i.e., 0 h immersed in PBS) and serves as a standard for comparative analysis. Compared to CZGO, CZGO-0 had good solubility in the PBS solution, as shown in Figure 1. CZGO-0 remained dispersed in water, whereas CZGO immediately settled at the bottom.

### 2.5. Immersion of CZGO-0 in the PBS Solution

An amount of 0.02 g of CZGO-0 was added into separate vials containing 20 mL PBS. The vials containing CZGO-0 in PBS were incubated in an incubator shaker (Excella E24 Incubator Shaker, New Brunswick^TM^, Marshall Scientific, Hampton, NH, USA) at 37 °C, at 170 rpm. The vials were retrieved from the shaker after pre-determined time intervals of 6, 18 and 48 h. The powders were isolated through centrifugation at 8000 rpm for 10 min, then dried overnight. The samples of interest are denoted as CZGO-6, CZGO-18 and CZGO-48, corresponding to the number of hours spent immersed in PBS, and will be comparatively studied with CZGO (i.e., a pristine starting material after annealing) and CZGO-0 (i.e., CZGO after surface hydroxylation).

### 2.6. Characterization

The morphology of the samples was characterized using transmission electron microscopy (TEM, Philips CM10 TEM, Philips Electronics, Eindhoven, The Netherlands). Before imaging, the samples were ultrasonically dispersed in 95% EtOH for 5 min and placed onto a Cu grid where TEM was carried out at an accelerating voltage of 60 kV. Energy-dispersive X-ray (EDX, Oxford Aztec X-Max50 SDD X-ray analyzer, Cambridge, MA, USA) analysis was performed using an EDX spectrometer attached to a scanning electron microscope (SEM, Hitachi SU3500 Variable Pressure SEM, Hitachi, Tokyo, Japan) to evaluate the elemental composition of the immersed samples. X-ray photoelectron spectroscopy (XPS) measurements were performed using the Kratos AXIS Supra (Kratos Analytical, Manchester, UK). All spectra were charge-corrected using the peak for adventitious carbon at 284.8 eV. Each scan was performed for 60 s, with 30–60 scans depending on the element of interest. Fourier-transform infrared (FT-IR) spectroscopy was conducted using a Bruker Alpha II spectrometer (Milton, ON, Canada). Atomic absorption spectroscopy (AAS) was used to evaluate the Zn concentration in PBS after the immersion test, using the Thermo Scientific iCE 3000 Series AA Spectrometer (London, ON, Canada). The diffraction patterns of the samples were obtained by X-ray diffraction (XRD, Inel CPS Powder diffractometer, Caltech, CA, USA, Cu Kα tube source, λ = 1.5406 Å). The photoluminescence (PL, AvaSpec-ULS2048XL-EVO, Avantes, Lafayette, CO, USA) profile of the samples was obtained by using a CCD spectrometer. The excitation source was a 254 nm hand-held flashlight (6 W). To ensure consistency between each PL measurement, the samples were placed onto a flat surface and spread out uniformly across a 2 mm × 2 mm area, with a consistent thickness. The distance between the light probe (optical fibre) and the sample was kept constant at 10 mm. The PersL lifetime was examined using an Acton SP2300i spectrograph (Teledyne Princeton Instruments, Acton, MA, USA) coupled with an CCD camera (iDUS401aBR-DD, Andor Technologies, Belfast, UK). A primary limestone glass-based homemade cylindrical cell with a flat and transparent window was used to ensure maximum light transparency. The analytes were loaded into the cell and positioned directly on top of the spectrometer, with a slit width of 1.5 mm. Before each measurement, the sample was excited using a 254 nm hand-held UV light (6 W) for 10 s. The integration time for each spectrum was set to 1 s, and the bi-exponentially fitted decay curve was obtained by plotting the maximum luminescence at 694.5 nm against time in seconds.

## 3. Results and Discussion

### 3.1. Morphology

**Figure 2:**
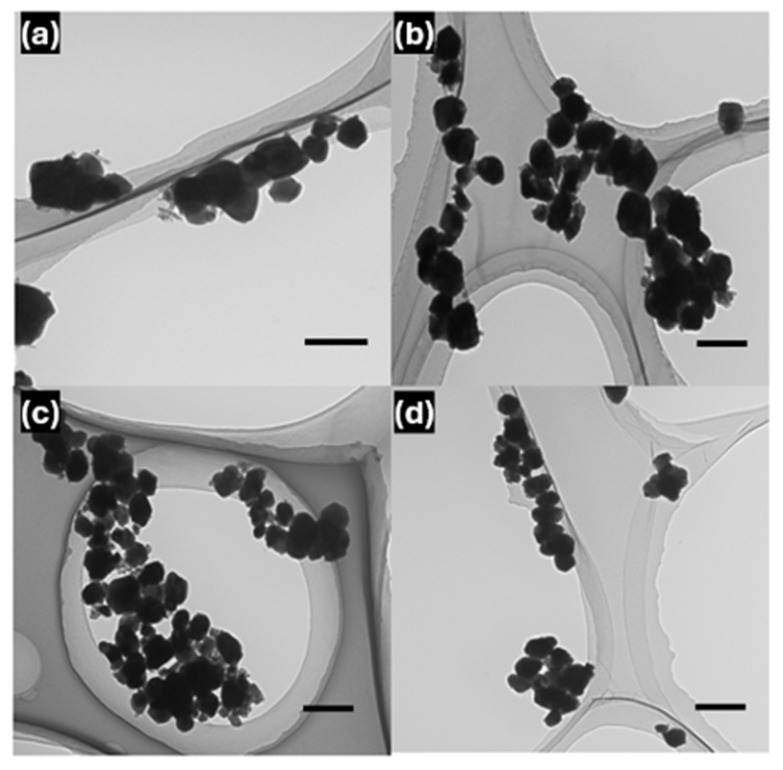
Transmission electron microscopy (TEM) images of (**a**) CZGO-0, (**b**) CZGO-6, (**c**) CZGO-18 and (**d**) CZGO-48. The scale bars in all figures are 200 nm.

The morphologies of CZGO-0 and the PBS-immersed samples were examined using TEM imaging and are displayed in Figure 2a–d. Compared to pristine CZGO (Figure S1a), CZGO-0 and the immersed samples do not show significant morphological changes after surface hydroxylation and short-term immersion in PBS. The size distribution plots of CZGO and the immersed samples are shown in Figure S1b–e, revealing that all sample groups have a mean NP diameter ranging from 80 to 100 nm. There is a difference in NP diameter as the immersion duration increases; however, there is a deviation between the sample groups. As a result, a standard two-tailed *t*-test was performed, which is a reliable method of statistical analysis to verify the significance of the observed mean diameter reduction [40]. A *p*-value sample calculation is shown in the Supplementary Materials. As shown in Table 1, when comparing the mean diameter of CZGO-0 with CZGO-6, a *p*-value of 0.08 is obtained, meaning there was no statistical relevance. Conversely, CZGO-18 and CZGO-48 had *p*-values of less than 0.05, indicating that the change in particle size is statistically significant. Therefore, although at a slow rate, CZGO-0 underwent dissociation and released ions during prolonged PBS immersion. We will return to this point later.

### 3.2. Luminescence Properties

**Figure 3:**
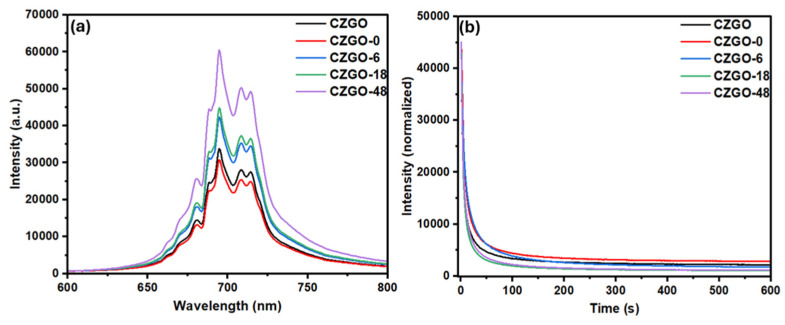
(**a**) Photoluminescence (PL) spectra and (**b**) normalized persistent luminescence (PersL) decay lifetime of CZGO and CZGO-0 after different PBS immersion durations. A 254 nm ultraviolet (UV) flashlight was used as the excitation source. The PersL decay was measured by monitoring the intensity of the 694.5 nm PL after the samples were charged under the UV light for 10 s.

Figure 3a shows the PL intensity of the CZGO-0 after immersion in PBS for various durations. Pristine CZGO before surface hydroxylation was also included. The samples were collected from the PBS solution and measured in powder form. All samples exhibited similar PL profiles, which match the characteristic Cr^3+^ emission of CZGO. The dominant N2 line at 694.5 nm represents Cr^3+^ adjacent to a cationic antisite defect (i.e., Zn^2+^ occupying the octahedral Ga^3+^ site or Ga^3+^ occupying the tetrahedral Zn^2+^ site). This defect has been related to the charge-trapping and recombination processes, both of which have been associated with the PersL mechanism of CZGO [41]. The zero-photon R line at 688 nm is ascribed to Cr^3+^ sitting in an unperturbed octahedral environment, indicative of successful Cr^3+^ doping into the octahedral Ga^3+^ site. The remaining features at 660–680 nm and 708–730 nm are due to anti-Stokes and Stokes phonon sidebands, respectively.

After surface hydroxylation, we notice a slight decrease in PL intensity compared to pristine CZGO, but with no noticeable alteration to the emission line profiles. After immersing CZGO-0 in PBS, a gradual enhancement in luminescence intensity was observed. The luminescence increase peaked at 48 h and, as demonstrated in Figure S2, prolonged exposure past 48 h did not significantly change the luminescence intensity. To eliminate the effect of the PBS solution, the PL measurements were repeated after the samples were collected from the PBS and redispersed in deionized water. As shown in Figure S3, a similar trend was observed, confirming that the PL enhancement originated from the CZGO NPs. On the other hand, immersing CZGO-0 in pure water did not result in any noticeable change in the PL intensity (Figure S4). This suggests that the surface of the CZGO-0 underwent interactions with the ions in the PBS, and these interactions, which resulted in a luminescence improvement, reached an equilibrium at around 48 h of immersion.

The improved optical properties after immersing CZGO in PBS is a significant finding. In previous studies, different bioimaging probes have been immersed in simulated physiological environments. However, those probes typically led to a decrease or non-noticeable change in luminescence over time. Zhou et al. studied the renal clearance of AuNPs and observed no significant changes to the luminescence after 48 h of exposure to fetal bovine serum [42]. In another study, Calderón-Olvera et al. evaluated the changes to the luminescence intensity and lifetime of another PersL material, Mn^2+^-doped ZnGeO_4_ NPs, after its immersion in PBS for 7 days [43]. Similarly, they did not observe any notable changes to the luminescence intensity or lifetime. To further elucidate the reason behind the improved luminescence properties, we analyzed the PL and PersL decay in greater detail.

The origin of the PersL from CZGO has been attributed to the presence of antisite (bulk) defects, and there have been studies that reported that the amount of antisite defects alters the intensity ratio of the N2 and R-line peaks in the PL spectra [41]. To address whether the immersion in PBS leads to any change in the antisite defect configurations in the CZGO-0, the PL spectra were normalized to the intensity of CZGO-48 at 694.5 nm. Shown in Figure S5, there was no change in the overall PL profile, indicating immersion in the PBS did not result in a change in the bulk defect structure. On the other hand, there are a few previous reports on the surface passivation of Cr-doped Zn gallogermanate, such as crystalizing the amorphous surface through thermal annealing or coating the particle surface with SiO_2,_ which demonstrated effectiveness in enhancing the PL intensity and extending the PersL lifetime [44]. The increase in PL intensity observed in our case might also be due to the structural modification on the particle surface.

Figure 3b shows the biexponentially fitted PersL decay curve for CZGO, CZGO-0 and the immersed samples. The intensity values were obtained from the PL maxima (694.5 nm), and the spectra were normalized to the same intensity to visualize the difference between their decay processes. Their lifetime is displayed in Table 2 and expressed using a bi-exponential function, as shown by Equation (1):

(1) $$I=I_{0}+\alpha_{1}\exp\left(\frac{t}{\tau_{1}}\right)+\alpha_{2}\exp\left(\frac{t}{\tau_{2}}\right)\;$$

where *I* is the intensity, *I*_0_ is the asymptotic constant offset, *α*_1_/*α*_2_ are the amplitudes, *t* is the time and *τ*_1_/*τ*_2_ are the decay times for the exponential components, respectively. The detailed comparison between the experimental and fitted spectra for each sample can be found in the Supporting Information Figure S6. There have been some debates on which exponential function to use when measuring the decay rate of PersL materials, as represented by different studies [45,46,47,48,49]. In this work, we generalized the fitted curve into two processes, the fast and the slow, which correspond to the shallow and deep electron traps, respectively. The fast component represents the immediate emission observed, which corresponds to luminescence caused by thermal stimulation. Conversely, the slower component stems from deeper traps, which are responsible for the longer, persistent emission of PersL materials originating from electrons that leave deep traps via quantum tunnelling [45].

It is worth mentioning that there are slight discrepancies between the luminescence intensity observed in PL and the initial intensity of the decay curve. To avoid saturating the detector, the start time of the decay measurement was set at 1 s after turning off the excitation source. As a result, we normalized their intensities at *t* = 1 s to evaluate the differences between their lifetimes. Although CZGO and CZGO-0 exhibited similar PL intensities under excitation, the former decayed faster. On the contrary, the samples immersed in PBS had larger amplitudes for both the fast and slow components, as reflected by the stronger starting intensity for CZGO-18 and CZGO-48. CZGO-18 and CZGO-48, which had the strongest initial luminescence, also had the fastest decay. This indicates that the structural modifications that influenced the initial luminescence intensity do not directly correlate with prolonging emission lifetime.

### 3.3. Surface Chemistry and Crystal Structure

**Figure 4:**
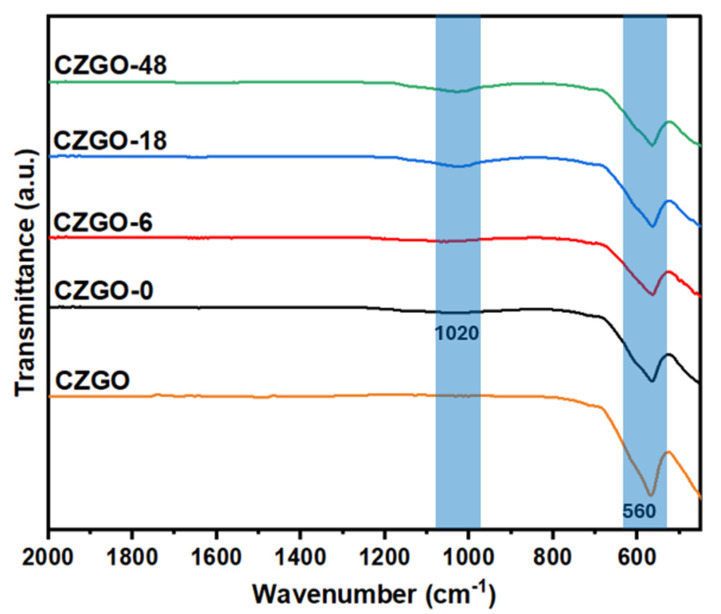
Fourier-transformed infrared (FT-IR) spectra of CZGO, CZGO-0 and PBS-immersed samples.

To explain the optical improvements after immersing CZGO-0 in PBS, the chemical structure of the samples was comparatively analyzed. Figure 4 shows the FT-IR spectra of the immersed samples in comparison to CZGO and CZGO-0. All samples have the characteristic Zn-O feature at ~560 cm^−1^, whereas the signature Ga-O stretch (typically found at ~450 cm^−1^) is beyond the working range of the FT-IR spectrometer used in this study [50]. After treatment with dilute HCl, CZGO-0 shows the emergence of a peak around 1020 cm^−1^, which is characteristic of the Zn-O stretch in ZnO [51,52]. This feature became more prominent at prolonged PBS exposure.

As a result of the suggested formation of ZnO, the elemental composition of CZGO-0 after PBS immersion was investigated. EDX analysis was performed to investigate whether the ion dissociation leads to a change in the elemental composition of the CZGO NPs. Table 3 summarizes the atomic ratios of the Ga/Zn of CZGO-0 after different immersion durations compared to CZGO-0. After hydroxylation, there was a noticeable decrease in Ga concentration relative to Zn. We attribute this deviation to the higher reactivity towards HCl by Ga^3+^ compared to Zn^2+^. Ga^3+^ is a stronger Lewis acid and would have a higher tendency to engage in interactions with the chloride ions in HCl. Furthermore, the presence of excess Zn may be in the form of ZnO, as evidenced by the FT-IR spectra.

There have been two common methods used to form hydroxylated surfaces on CZGO and CZGO-like NPs. The first method involves wet-grinding and dispersing the CZGO NPs in dilute HCl [22,33,34,53,54], while the other method involves dispersing the NPs in dilute NaOH [20,55,56,57]. To the best of our knowledge, the effectiveness of the two methods has not been comparatively studied. Figure S7 compares the luminescence, solubility and dispersibility between the two treated samples. As shown, the luminescence of the two methods is comparable. However, CZGO treated with dilute HCl demonstrates better solubility and dispersibility. As a result, we decided to conduct the immersion test using the HCl-treated sample despite the notably lower Ga/Zn ratio.

**Figure 5:**
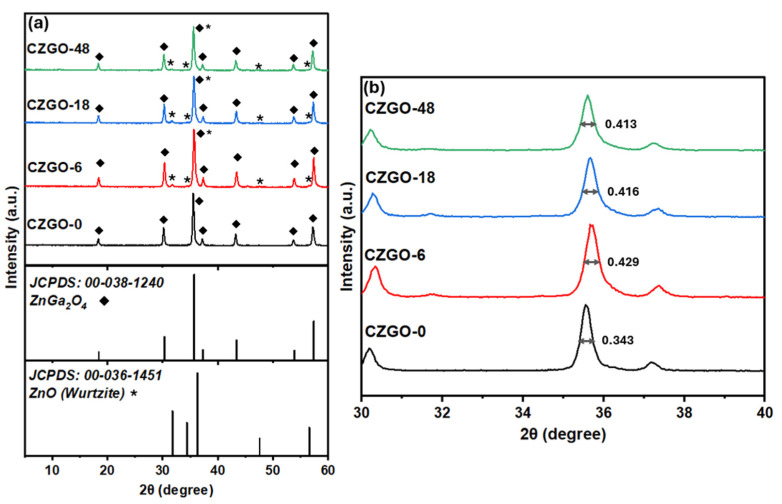
(**a**) The diffraction patterns of CZGO-0 and the immersed samples compared to the references ZnGa_2_O_4_ (JCPDS: 00-038-1240) and ZnO (JCPDS: 00-036-1451). The diamond and asterisk symbols indicate peaks corresponding to the ZnGa_2_O_4_ and ZnO (wurtzite) references, respectively. (**b**) The magnified region of diffraction pattern between 2θ = 30–40°. The full width at half maximum (FWHM) values of the highest intensity peak in CZGO were listed in the figure.

To verify whether the change in the Ga/Zn ratio influenced the crystal structure of CZGO, XRD was conducted, as shown in Figure 5. Figure 5a shows the X-ray diffraction pattern of the immersed samples in comparison to a reference ZnGa_2_O_4_ (JCPDS: 00-038-1240). Firstly, the diffraction patterns of the immersed samples closely resemble CZGO-0 and the reference ZnGa_2_O_4_, indicating prolonged immersion did not alter the crystallinity of the NPs. However, we noticed the appearance of several additional peaks at 2θ = 32, 34.3, 47.6 and 56.6°, which match the diffraction pattern of ZnO. A zoomed-in view of the 2θ between 30° and 40° is shown in Figure 5b. As shown, the full width at half maximum (FWHM) of the highest intensity peak in CZGO increases after treatment with HCl. Notably, the ZnO-related peaks cannot be observed in CZGO-0 despite the low Ga/Zn ratio shown earlier. This suggests the formation of amorphous ZnO upon treating the CZGO in diluted HCl, which is eventually crystallized during the immersion process.

**Figure 6:**
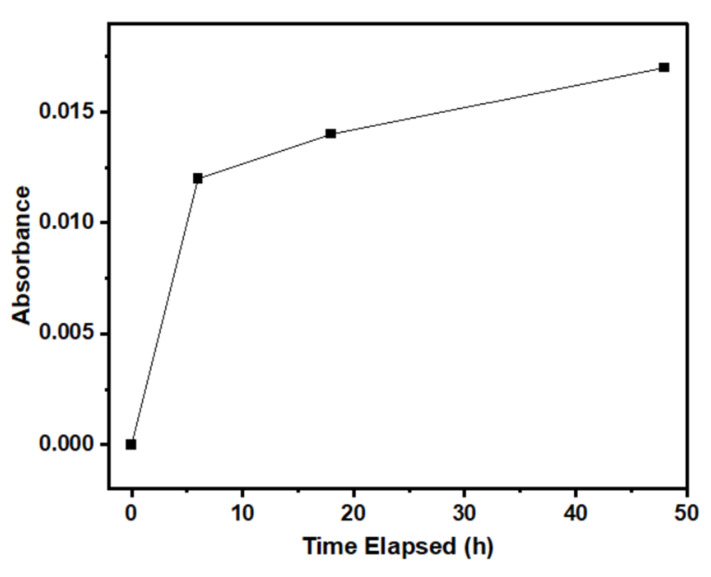
Atomic absorption spectra (AAS) of PBS solution before and after immersing 20 mg of CZGO-0 in 20 mL of PBS.

Figure 6 shows the rate of Zn^2+^ release measured using AAS. A Zn^2+^ standard was created by dissolving 4.56 mg of Zn(NO_3_)·6H_2_O in 100 mL deionized water. The PBS solution without exposure to CZGO-0 was also measured. Since pure PBS does not contain any Zn components, no Zn^2+^ was detected. After 6 h of immersing CZGO-0 in PBS, the absorbance of the solution quickly rose to 0.012, which corresponds to 0.2 ppm of Zn^2+^. The concentration of Zn^2+^ in the solution positively correlates to the time CZGO-0 spent in the PBS solution. After 48 h of immersion, the Zn^2+^ in the PBS was found to be around 0.3 ppm. Considering the increase in Ga/Zn ratio throughout the immersion process, this confirms the release of Zn^2+^ ions by ZnO and CZGO. The dissociation of CZGO into Zn^2+^ ions was also observed in our previous work: when CZGO was involved in the formation of a CZGO–calcium phosphate composite and Zn^2+^ was found doped into the calcium phosphate [58]. The AAS results in the current study provide direct evidence confirming the high activity of Zn^2+^ in the CZGO NPs.

**Figure 7:**
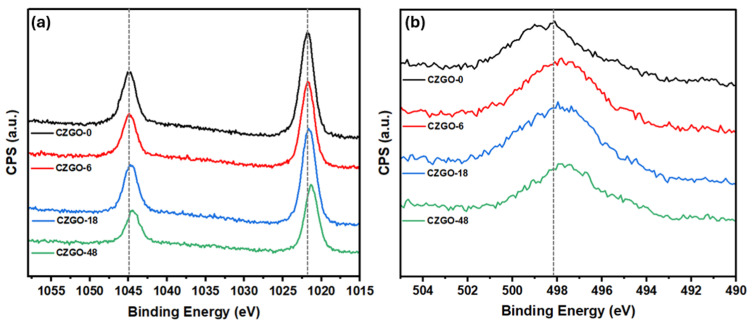
(**a**) X-ray photoelectron spectroscopy (XPS) spectra of the Zn 2p region and (**b**) Zn LMM region.

XPS was conducted to investigate the electronic structure of Zn. Figure 7a,b shows the XPS spectra of the Zn 2p and Zn LMM regions, respectively. The wide scan can also be found in Figure S8. Each Zn 2p spectrum contains both the Zn 2p_1/2_ and 2p_3/2_ peaks, and the distance between the two peaks has been used to identify the oxidation state of Zn in a given compound [59]. The peak separation for all samples between the Zn 2p_1/2_ and 2p_3/2_ approximates 23.15 eV, which corresponds to the divalent of zinc ion and is consistent with CZGO synthesized in the literature [60]. The subtle peak shift observed in the XPS spectra is within the calibration error (~0.3 eV) [61]. Furthermore, the Zn 2p signals overlap with the O KLL peaks when probed using an Al Kα source [62]. As a result, it is more reliable to utilize the calculated Zn Auger parameter (the sum of the binding energy of Zn 2p_3/2_ and the kinetic energy of Zn LMM) for species identification [59]. As shown in Table 4, the Zn Auger parameters are shown to decrease from CZGO-0 to CZGO-48. We attribute the change in binding energy to the formation of ZnO. Conversely, each Ga 2p spectrum has a peak separation of 27 eV (Figure S9), which indicates the presence of trivalent gallium [60]. The peak shift observed in the Ga XPS spectra is within the calibration error, indicating no observable change in the Ga electronic structure.

### 3.4. Proposed Formation Mechanism of ZnO

The results obtained from the XPS spectra reinforce the idea that ZnO is present after immersing CZGO-0 in PBS. Moezzi et al. previously reported a comprehensive analysis of the different aqueous pathways for the formation of ZnO NPs [63]:

(2) Zn^2+^_(aq)_ + OH^−^_(aq)_ → Zn(OH)^+^_(aq)_

(3) Zn(OH)^+^_(aq)_ + OH^−^_(aq)_ → Zn(OH)_2(s)_

(4) Zn(OH)_2(s)_ ⇌ Zn(OH)_2(aq)_

(5) Zn(OH)_2(aq)_ → ZnO_(s)_ + H_2_O_(l)_

(6) Zn(OH)_2(s)_ → ZnO_(s)_ + H_2_O_(l)_

where these reactions were shown to be governed by the pH of the solution. Equations (2) and (3) describe the formation of zinc hydroxide complexes from Zn^2+^. In neutral to slightly basic conditions, most Zn will be present as solid Zn(OH)_2_ and ZnO. It is worth noting that the luminescence increase was only observed when CZGO-0 was immersed in the PBS solution. As mentioned earlier, the immersion of CZGO-0 in water did not result in a noticeable increase in luminescence. The immersion of CZGO, which had a higher Ga/Zn ratio, also did not lead to notable improvements (Figure S10).

We hypothesize that the luminescence enhancement is tied to the formation of ZnO. After surface hydroxylation, CZGO-0 was shown to be Ga-deficient, which is evident from the EDX data. The FT-IR, XRD and XPS analyses support the formation of ZnO after HCl treatment. In the PBS solution, NaH_2_PO_4_ and KH_2_PO_4_ act as the conjugate acid and base, respectively. Throughout the immersion process, Zn^2+^ ions, which are acidic, are gradually released in the PBS. In typical neutral buffers, HPO_4_^2−^ ions may interact with the Zn^2+^ ions to maintain the pH. As a result, the stable pH and ionic conditions maintained by the PBS solution can result in a higher-order crystallization process by ZnO. It is suspected that the surface formation of ZnO on the CZGO surface leads to improved luminescence due to the partial suppression of surface defects.

The increase in luminescence observed when immersing CZGO NPs in PBS solution is particularly noteworthy. As previously mentioned, it is uncommon to see such an enhancement after exposure to physiological media. While no change in luminescence generally does not pose an issue, some phosphors have shown a reduction in luminescence under similar conditions, which limits their potential for the intended bioimaging applications. Our systematic study on the short-term immersion of CZGO NPs in PBS demonstrates a significant improvement in luminescence, suggesting the material can be a promising candidate for bioimaging applications that take place within several hours to a few days. For example, the bioimaging of tumour-bearing mice occurred within 14 h, whereas the visualization of food-borne toxins in a separate study took place over 24 h [21,30]. The results observed in our immersion test can potentially improve pre-existing applications while also opening new perspectives for biomedical imaging in physiological environments.

## 4. Conclusions

In this study, hydroxylated CZGO was immersed in PBS for up to 48 h to evaluate the short-term optical/structural changes upon its exposure to physiological media. Interestingly, a significant improvement in the luminescence intensity and lifetime of CZGO was observed when it was exposed to the PBS solution, which is uncommon among optical nanomaterials for bioapplications. The chemical structure of the immersed samples was comparatively analyzed through several structure elucidation techniques. The FT-IR and EDX analyses suggested the formation of ZnO upon surface hydroxylation using dilute HCl, which, to the best of our knowledge, has not previously been reported. The XRD revealed that the ZnO is amorphous until its immersion in PBS. XPS verified the formation of ZnO, revealing the co-existence of ZnO alongside CZGO. The enhancement in optical properties is attributed to the formation and crystallization of surface-adhered ZnO, which allows for the passivation of surface defects. These findings showcase the advantages of hydroxylation as a simple surface modification technique. While this study demonstrated an improvement in the luminescence properties of CZGO NPs after exposure to the PBS solution, it is essential to assess the change in luminescence behaviour upon immersion in different physiological environments. The buffered solution used in this study was designed to replicate the pH and ionic concentration of human blood, but the various organs in the body have distinct physiological conditions, which can be further altered upon the occurrence of a disease. Therefore, additional research on CZGO NPs under more complex physiological conditions would provide valuable insights for the biomaterials community. In this work, PersL NPs with improved hydrophilicity and luminescence prove to be a promising candidate for bioimaging applications.

## Figures and Tables

**Table 1:** The *t*-test results comparing the mean particle sizes of CZGO-6, CZGO-18 and CZGO-48 with CZGO-0. *p* < 0.05 indicates a statistical significance between the mean particle sizes of the sample groups.

Sample	Mean Particle Size (nm)	Comparison of Time	*p*-Value
CZGO-0	97 ± 23	-	-
CZGO-6	91 ± 19	0 ➔ 6 h	0.09
CZGO-18	87 ± 21	0 ➔ 18 h	0.03
CZGO-48	87 ± 22	0 ➔ 48 h	0.02

**Table 2:** Persistent luminescence (PersL) decay fit parameters of CZGO after different immersion durations. *α*_1_ and *α*_2_ represent the amplitudes of the fast and slow decay processes, respectively. *τ*_1_ and *τ*_2_ represent the decay times (in seconds) for the exponential components, respectively.

Sample	*α*_1_	*α*_2_	Lifetime (s)	
			*τ*_1_	*τ*_2_
CZGO	20,309.89	3783.79	3.76	45.04
CZGO-0	14,001.08	2807.37	6.22	59.24
CZGO-6	26,659.41	7738.26	6.15	60.02
CZGO-18	47,945.92	8179.55	3.60	37.10
CZGO-48	41,436.63	9147.23	4.02	38.93

**Table 3:** The Ga/Zn ratios of CZGO, CZGO-0 and the PBS-immersed samples. The values were derived from the energy-dispersive X-ray (EDX) measurement.

	CZGO	CZGO-0	CZGO-6	CZGO-18	CZGO-48
Ga/Zn	2.16 ± 0.05	1.48 ± 0.17	1.60 ± 0.13	1.58 ± 0.14	1.76 ± 0.06

**Table 4:** The binding energy (B. E.) of Zn 2p_3/2_, the kinetic energy (K. E.) of the Zn LMM and the Zn Auger parameter.

Sample	B. E. (eV)	K. E. (eV)	Auger Parameter (eV)
CZGO-0	1021.73	988.5	2010.23
CZGO-6	1021.66	989.0	2010.66
CZGO-18	1021.49	989.0	2010.49
CZGO-48	1021.18	989.4	2010.58
CZGO	1021.53	988.4	2009.93
ZnO [59]	1021.00	989.4	2010.40

## Data Availability

The data presented in this study are available upon request from the corresponding author.

## Supplementary Materials

The following supporting information can be downloaded at: https://www.mdpi.com/article/10.3390/nano15030247/s1, Synthesis of CZGO-96 and CZGO-7Day; Synthesis of CZGO-NaOH; Sample calculation for *p*-value using two-tailed *t*-test; Figure S1: (a) TEM image of CZGO and size distribution (N > 80) plots of (b) CZGO-0, (c) CZGO-6, (d) CZGO-18 and (e) CZGO-48; Figure S2: (a) Photoluminescence spectra of the samples of interest, including CZGO-96 and CZGO-7Day. (b) Luminescence intensity at 694.5 nm at different immersion times; Figure S3: Solution PL of CZGO-0 and immersed samples redispersed in deionized water; Figure S4: PL spectra of CZGP-0, CZGO-48 and CZGO-Water. A 2564 nm UV flashlight was used as the excitation source. CZGO-Water was made by immersion CZGO-0 in deionized water for 48 h; Figure S5: PL spectra of CZGO-0 and the PBS-immersed samples. All spectra normalized to the maximum PL intensity in CZGO-48. Figure S6: The experimental data and bi-exponentially fitted PersL decay curve for CZGO, CZGO-0 and the PBS-immersed samples; Figure S7: The difference in dispersion between CZGO-0 and CZGO-NaOH in water after (a) 2 min and (b) 5 min. (c) CZGO-0 and CZGO-NaOH under 254 nm excitation. (d) PL spectra of CZGO-0 and CZGO-NaOH; Figure S8: The wide-scan XPS spectra of CZGO-0 and the PBS-immersed samples; Figure S9: (a) The XPS spectra of the Ga 2p region and (b) Ga LMM region; Figure S10: PL spectra of CZGO and non-hydroxylated (NH) CZGO immersed in PBS for 6, 18 and 48 h.
