# Supplementary material for: Enhanced Persistent Luminescence from Cr3+-Doped ZnGa2O4 Nanoparticles upon Immersion in Simulated Physiological Media
Source: Nanomaterials (Basel). 2025 Feb 6;15(3):247. doi: 10.3390/nano15030247 (PMC11820434; doi:10.3390/nano15030247)
Supplement: Supplementary file 1 [file nanomaterials-15-00247-s001.zip › nanomaterials-3443915-supplementary.pdf]

Supporting Information

Enhanced Persistent Luminescence from Cr^{3+}-Doped $ZnGa_2O_4$ Nanoparticles upon Immersion in Simulated Physiological Media

Clement Lee, David Park, Wai-Tung Shiu, Yihong Liu and Lijia Liu *

Department of Chemistry, Western University, 1151 Richmond Street, London, ON N6A 5B7, Canada; hlee752@uwo.ca (C.L.); dpark68@uwo.ca (D.P.); wshiu@uwo.ca (W.-T.S.); yliu3465@uwo.ca (Y.L.)

* Correspondence: lijia.liu@uwo.ca

Synthesis of CZGO-96 and CZGO-7Day:

0.02 g of CZGO-0 was added to a vial containing 20 mL PBS. The vials containing CZGO-0 in PBS were incubated in an incubator shaker (Excella E24 Incubator Shaker, New Brunswick) at 37 °C, at 170 rpm. The vials were retrieved from the shaker after pre-determined time intervals of 96 hours and 7 days. The powders were isolated through centrifugation at 8000 rpm for 10 minutes, then dried overnight. The samples of interest are denoted as CZGO-96 and CZGO-7Day (i.e., corresponding to hours spent immersed in the phosphate-buffered saline) and will be comparatively studied with CZGO, CZGO-0 and the short-term immersed samples.

Synthesis of CZGO-NaOH

First, 0.005 g of CZGO NPs were placed in a mortar, followed by 0.5 mL of 5 mM NaOH. The powder was wet-grinded for 15 minutes and placed in a beaker containing an additional 4.5 mL of 5 mM NaOH. The solution was stirred in the beaker overnight and collected by centrifugation at 8500 rpm for 10 minutes. The product was purified by washing the powder in isopropanol and water at 8500 rpm for 10 minutes twice.

Particle size analysis details

To obtain the *p*-value on the particle size of a selected pair of samples, a two-tailed *t*-Test was performed. Following the equation below

$$t = \frac{M_1 - M_2}{\sqrt{\frac{s_1^2}{n_1} + \frac{s_2^2}{n_2}}}$$

, where M is the average particle size, s^2 is the standard deviation, and N is the number of particles counted. The dataset with subscript 1 is the pristine sample (i.e. CZGO-0), and the one with subscript 2 was the immersed sample. Using the CZGO-6 as an example, the t value can be calculated as follows.

$$t = \frac{97.2 - 91.4}{\sqrt{\frac{23.3}{80} + \frac{19.5}{80}}}$$
$$t = \frac{5.8}{3.4}$$
$$t = 1.71$$

Due to the variance in sample size and standard deviations, the Welch-Satterthwaite equation is used to calculate for the degrees of freedom:

$$df = \frac{\left(\frac{s_1^2}{n_1} + \frac{s_2^2}{n_2}\right)^2}{\frac{\frac{s_1^2}{n_1}}{n_1 - 1} + \frac{\frac{s_2^2}{n_2}}{n_2 - 1}}$$

$$df = \frac{\left(\frac{23.3^2}{80} + \frac{19.5^2}{80}\right)^2}{\frac{\frac{23.3^2}{80}}{79} + \frac{\frac{19.5^2}{80}}{79}}$$

$$df = \sim 157$$

With the t-statistic of 1.71 and the degree of freedom of ~157, the *p*-value can be found using the Excel function: = T.DIST.2T(ABS(1.71), 157) to obtain a value of 0.089. As a result, there is no

statistical significance at the 0.05 level when comparing the mean diameters of CZGO-0 and CZGO-6, given their deviation and sample size.

The similar procedure was conducted to calculate all p values listed in Table 1.

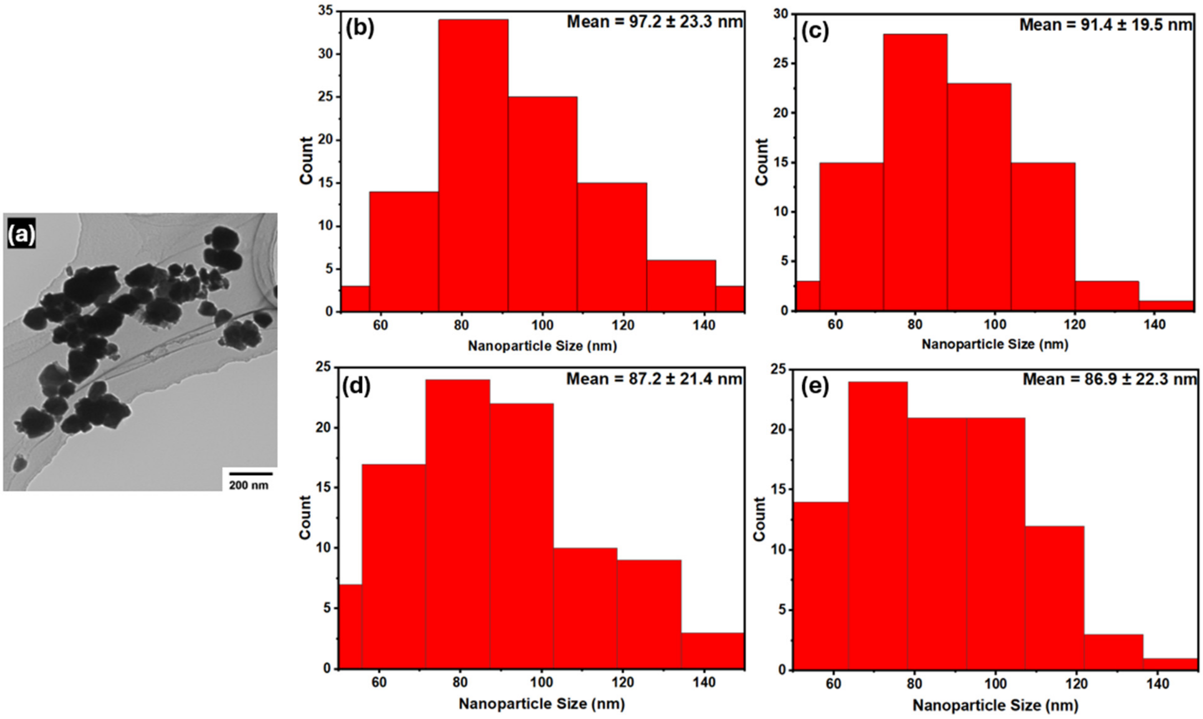

Figure S1. (a) TEM image of CZGO and size distribution (N > 80) plots of (b) CZGO-0, (c) CZGO-6, (d) CZGO-18, and (e) CZGO-48.

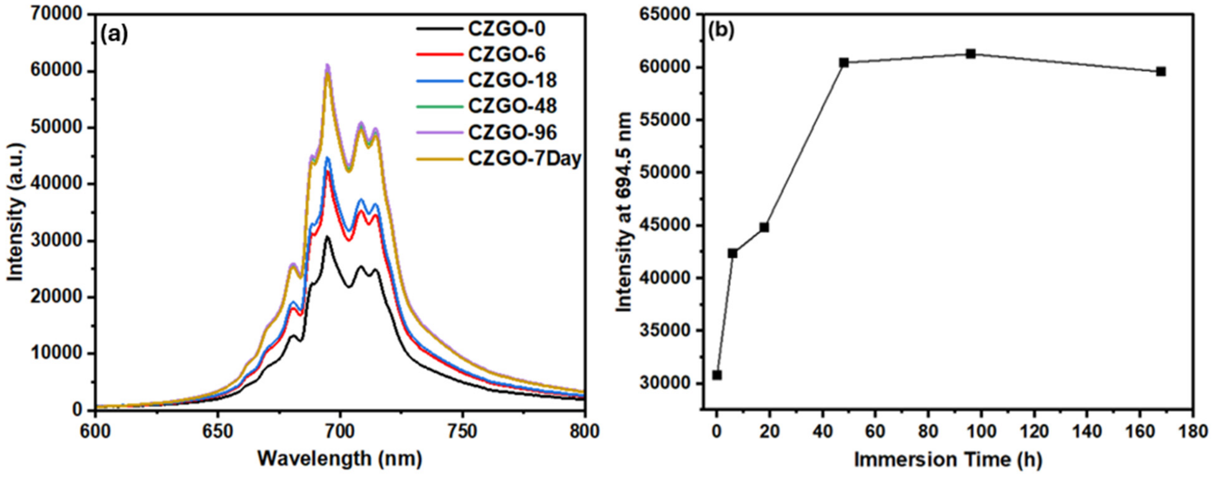

Figure S2. (a) Photoluminescence spectra of the samples of interest, including CZGO-96 and CZGO-7Day. (b) Luminescence intensity at 694.5 nm at different immersion times

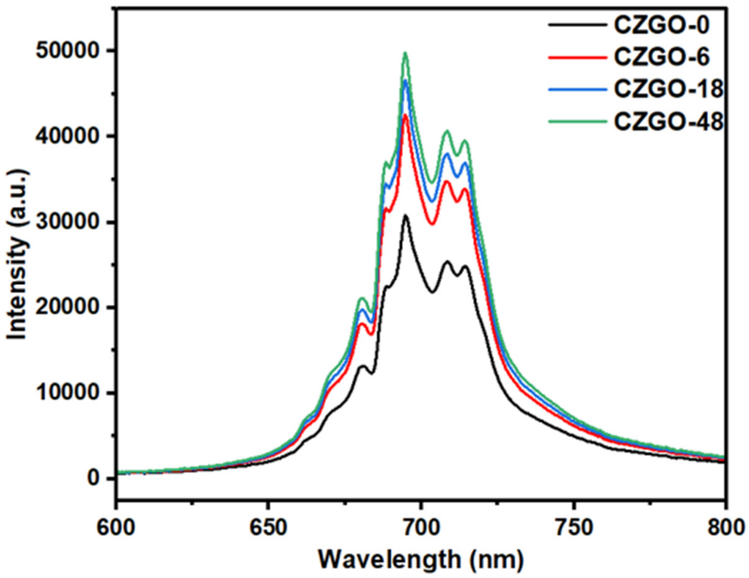

Figure S3. Solution PL of CZGO-0 and immersed samples redispersed in deionized water.

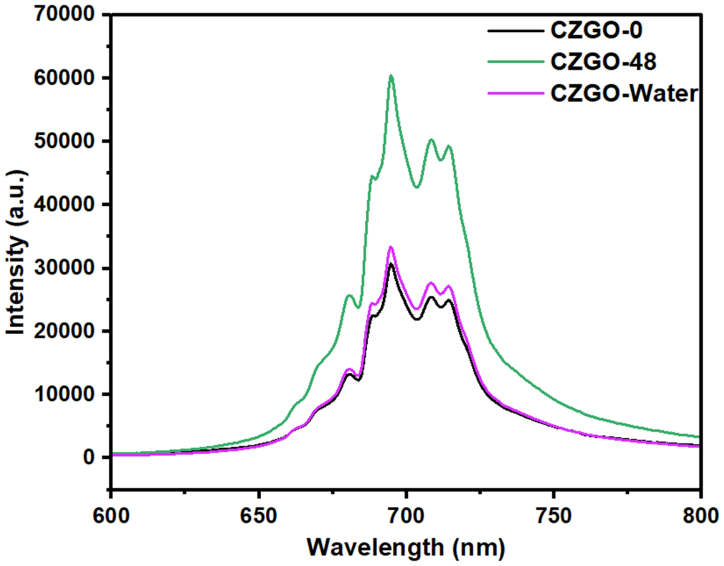

Figure S4. PL spectra of CZGO-0, CZGO-48 and CZGO-Water. A 254 nm UV flashlight was used as the excitation source. CZGO-Water was made by immersing CZGO-0 in deionized water for 48 hours.

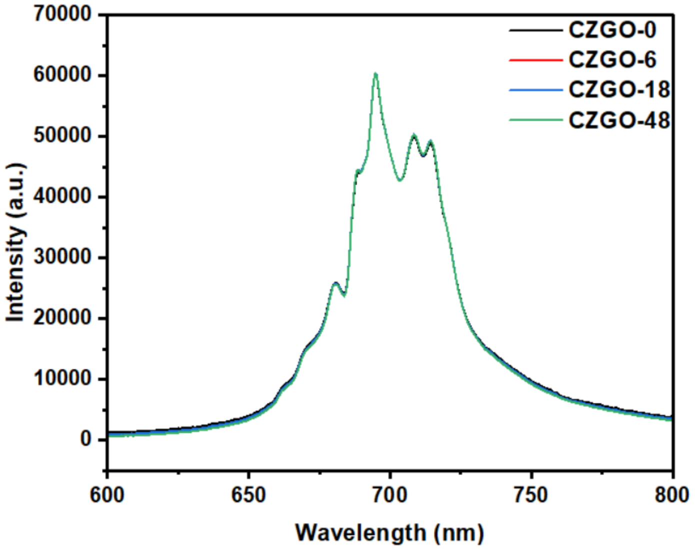

Figure S5. PL spectra of CZGO-0 and the PBS-immersed samples. All spectra normalized to the maximum PL intensity in CZGO-48.

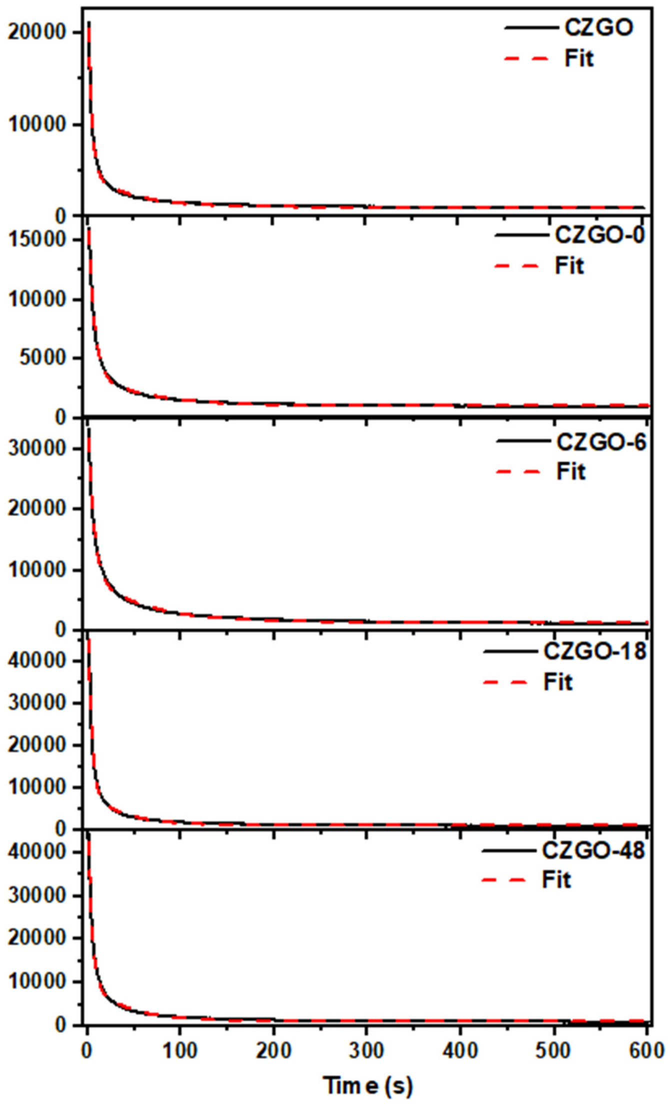

Figure S6. Experimental data and bi-exponentially fitted PersL decay curve for CZGO, CZGO-0 and the PBS-immersed samples.

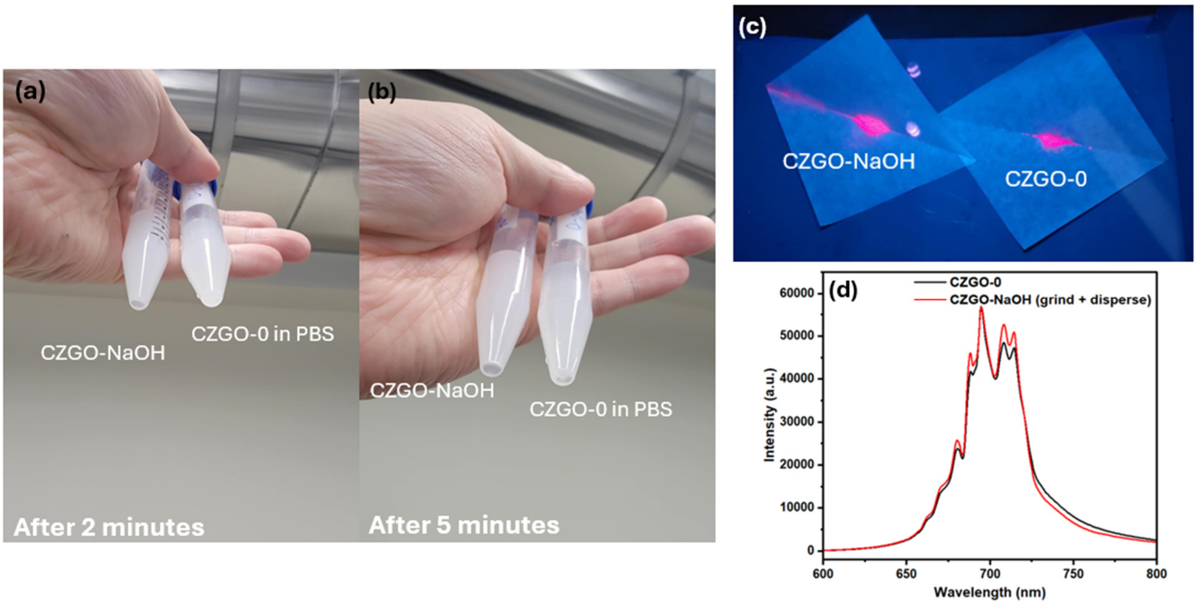

Figure S7. The difference in dispersion between CZGO-0 and CZGO-NaOH in water after (a) 2 minutes and (b) 5 minutes. (c) CZGO-0 and CZGO-NaOH under 254 nm excitation. (d) PL spectra of CZGO-0 and CZGO-NaOH.

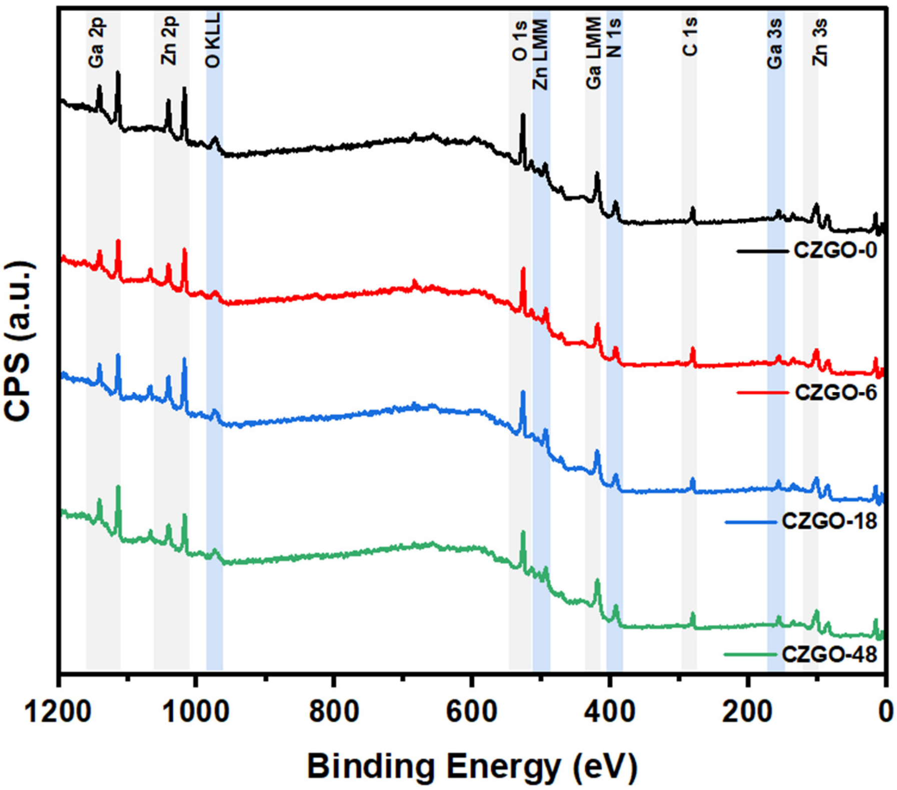

Figure S8: Wide scan XPS spectra of CZGO-0 and the PBS-immersed samples.

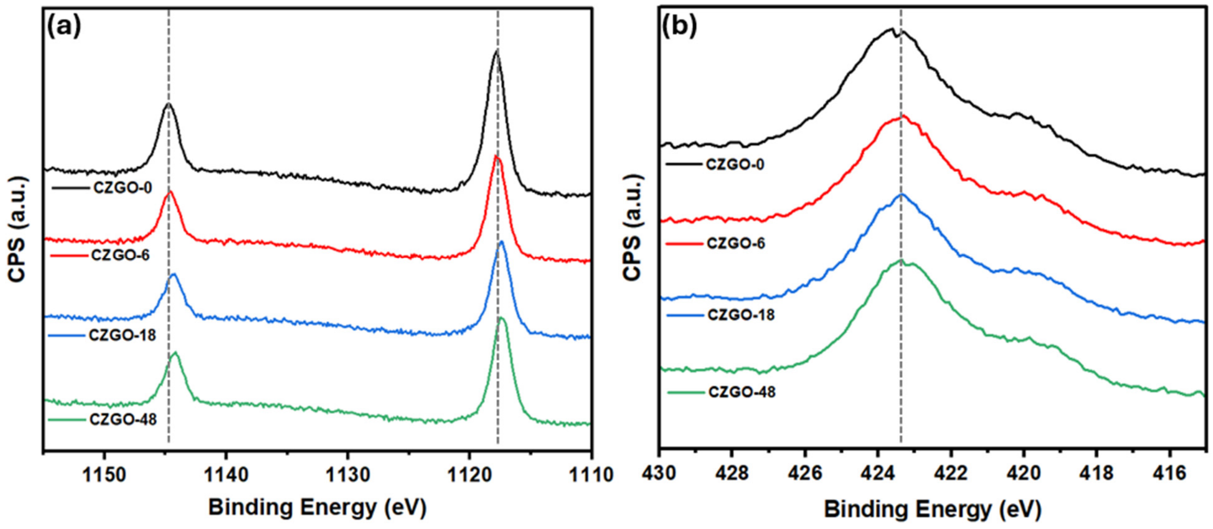

Figure S9. (a) XPS spectra of the Ga 2p region and (b) Ga LMM region.

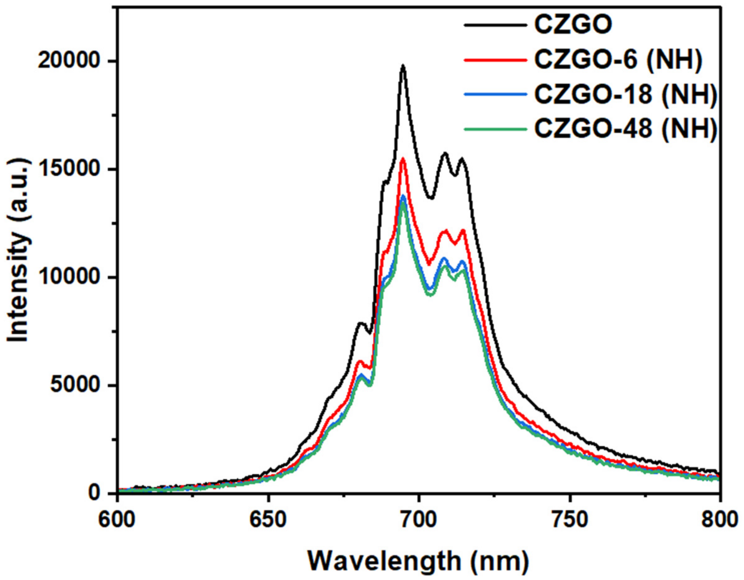

Figure S10. PL spectra of CZGO and non-hydroxylated (NH) CZGO immersed in PBS for 6, 18 and 48 hours.
